# Discospondylitis caused by *Brucella canis* in a dog imported to Sweden

**DOI:** 10.1186/s13028-026-00859-4

**Published:** 2026-04-09

**Authors:** Anna Bonnevie, Ruth Pleva, Tomas Jinnerot, Alexandra Leijon, Jennifer Spens, Andrés Álvarez Fernández, Emelie Pettersson, Bodil Ström Holst

**Affiliations:** 1 Department of Animal Health and Antimicrobial Strategies, Swedish Veterinary Agency, 751 89 Uppsala, Sweden; 2 Department of Clinical Sciences, Swedish University of Agricultural Sciences, Box 7054, 750 07 Uppsala, Sweden; 3 Department of Microbiology, Swedish Veterinary Agency, 751 89 Uppsala, Sweden; 4 Department of Pathology and Wildlife Diseases, Swedish Veterinary Agency, 751 89 Uppsala, Sweden; 5 Evidensia Specialist Animal Hospital Strömsholm, Djursjukhusvägen 11, 734 94 Strömsholm, Sweden; 6 Department of Epidemiology, Surveillance and Risk Assessment, Swedish Veterinary Agency, 751 89 Uppsala, Sweden

**Keywords:** Brucellosis, Canine, Cross-border, Infection, Infectious, Transport, Zoonosis, Zoonotic

## Abstract

**Background:**

*Brucella canis* is a zoonotic bacterium, causing mainly reproductive disorders and discospondylitis in dogs, but infections may also be subclinical. Mating is one of the main transmission routes, putting breeding dogs and stray dogs at risk of infection. Confirmatory diagnosis relies on positive culture or PCR. This is difficult to achieve due to intermittent and low-level bacterial excretion. Serological testing might give rise to both false negative and false positive results, making interpretation difficult. Since the first reported Swedish case 2011, the infection has been confirmed in a handful of cases, predominantly in breeding dogs. Several reports highlight the risk of brucellosis in dogs imported from Eastern Europe.

**Case presentation:**

This case study describes a one-year-old intact male dog originating from a dog shelter in Romania, which was presented to a veterinary hospital in Sweden with signs of pain of unknown origin. A suspicion of *B. canis* was raised based on clinical signs and history of the dog. Serological testing by enzyme-linked immunoassay for *B. canis* was positive. Resolution of clinical signs was not achieved despite treatment by robenacoxib and pregabalin. The dog was euthanised and sent for necropsy, showing a chronic multifocal prostatitis and a spondylosis-like, ossified change over L1–L2 in the lumbar spine. Infection was confirmed by positive result of two PCR tests, targeting the IS711 insertion sequence of *Brucella* spp. Four contact dogs were examined because of the confirmed infection. None of these dogs showed clinical signs of disease. Three of the contact dogs were positive on one or more test for *B. canis* antibodies. One of the seropositive contact dogs was euthanised and sent for necropsy. A mild generalised lymphadenitis was noted, but *B. canis* could not be confirmed.

**Conclusions:**

This is the first described Swedish case of *B. canis* in a non-breeding dog. The case highlights the complexity of canine brucellosis, including the array of clinical signs and the multiple diagnostic methods that may be necessary to confirm an infection. It also shows the risk of introducing infections through cross-border transportation of dogs.

## Background

*Brucella canis* is a gram-negative, facultative intracellular coccobacillus, with dogs as its primary host [1]. Since it was discovered in the 1960s in the United States of America (USA), as a causative agent of abortions in Beagle dogs, it has been recognised in many areas of the world [2].

### Clinical signs

In addition to abortions, infection can result in full-term stillbirth, as well as in birth of live pups, which frequently succumb shortly after birth, although survival has also been reported [1]. In males, epididymitis, orchitis and prostatitis are the main clinical signs. Chronically infected males often develop testicular atrophy and infertility due to poor sperm quality [3]. Infections with *B. canis* in dogs may also cause non-reproductive clinical signs, predominantly discospondylitis and uveitis [4–9]. Subclinical infections are likely common but underdiagnosed [10, 11].

### Transmission routes and bacterial shedding

Transmission occurs primarily through direct contact with body fluids, especially aborted material and reproductive fluids, which typically contain high bacterial loads, up to 10^8^ viable organisms/mL [10]. Excretion of bacteria in urine and other body fluids is lower and might be intermittent, indicating that longer contact time is necessary for transmission between individuals through this route [12]. Transmission via contaminated fomites, transplacental transfer, cutaneous inoculation and inhalation of aerosolised material is also possible [1, 10, 12].

### Diagnostic methods

Detection of *Brucella* spp. by bacterial culture or polymerase chain reaction (PCR) confirms the infection. However, after the initial bacteremic phase, the bacteria tend to localise intracellularly in reticuloendothelial cells and in the genital tract of males and pregnant females [10]. Especially in subclinically infected dogs, or in dogs with non-reproductive clinical signs, bacteria can be present only intermittently in samples chosen for culture. Hence, the sensitivity may be low and a negative culture or PCR may not rule out infection [11, 13]. Serological analysis is commonly used for diagnostic purposes and there are several methods for antibody detection, such as rapid slide agglutination test (RSAT), agar gel immunodiffusion test (AGID), enzyme linked immunosorbent assay (ELISA) and lateral flow immunoassay (LFIA) [11, 14, 15]. False positive serological results may occur, regardless of the chosen method, mainly due to cross reactions with other Gram-negative bacteria. False negative results may also occur due to fluctuating antibody levels or suboptimal timing of sampling [11, 13].

### Treatment and prognosis

Antibiotic treatment prior to sampling can also result in false negative cultures and serology results [11, 16]. Despite several proposed treatment protocols for dogs, clearance of the infection is not always achievable, resulting in relapse. Due to the common lack of an acceptable treatment response, and the fact that the infection is difficult to rule out because of low sensitivity and specificity of the available diagnostic tests, *B. canis* is usually regarded as non-treatable in dogs [1].

### Zoonotic potential and public health relevance

Like several other *Brucella* species, *B. canis* is a zoonotic agent, although scientific reports of human cases are scarce [16]. The symptoms in humans are generally non-specific, with undulating fever, chills and malaise, with more severe complications described in rare cases [16, 17]. Humans can be exposed to the bacteria either through direct contact with dogs, especially during or soon after abortion or parturition, or through laboratory exposure [18, 19]. There are no serological tests available that are intended for human samples, thus a diagnosis is made on a clinical suspicion confirmed by culture [16].

### Canine brucellosis in Sweden and lack of surveillance

The first case of *B. canis* in Sweden, clinically presented as an abortion in an imported breeding bitch, was reported in 2011 [20]. In 2013, three breeding dogs in another Swedish kennel were confirmed to be positive by culture [21]. These three dogs had also been imported or had been in close contact with an imported dog. Further, in 2018, *B. canis* was cultured and confirmed by PCR in a sample from a chronic fistulated wound in the prescapular area of an imported neutered female dog. The wound had been present for about three months according to the owner (personal communication, SVA).

### Global distribution and emerging European cases

Systematic surveillance and studies are lacking worldwide and the distribution of canine brucellosis due to *B. canis* is hence largely unknown [14, 16]. *Brucella canis* is considered endemic in South and Central America, and in the southern parts of the USA, and has been reported from several countries in Asia and Africa [11]. In Europe, an increasing number of cases has been reported in countries where the disease was previously not known, such as the United Kingdom (UK), Germany and the Netherlands. These cases are frequently associated with importation of dogs, particularly shelter dogs from Eastern European countries [14, 22–24]. Romania is a major source for canine exports within Europe, accounting for 32% (n = 999) of all imported dogs into Sweden in 2024 (personal communication The Swedish Board of Agriculture). The total yearly import from other EU countries ranges from 3000 to 6000 dogs, and additionally 500 to 1800 dogs are imported from countries outside the EU (personal communication The Swedish Board of Agriculture). These numbers include dogs imported privately as pets, as well as dogs from organisations focusing on re-homing shelter and stray dogs. The Swedish import statistics does not state the background of the dogs, but it is likely that many of them are shelter dogs with an unknown background, similar to the situation in the UK [24], thereby having an increased risk of being infected with *B. canis.* Lacking mandatory serological screening for canine brucellosis in Romania, the prevalence of infection among shelter dogs is not known, but one study on 100 shelter dogs found a seroprevalence of 8% [25]. As brucellosis is known to be more prevalent among strays and shelter dogs, cross-border sale of dogs from these populations might come with a risk of introducing *B. canis* to new areas [2, 14].

This case report describes a case of brucellosis in an imported dog with discospondylitis and includes pathological and histopathological findings. Serological and microbiological examinations of four contact dogs, as well as pathology findings of one of them, are also described.

## Case presentation

A one-year-old, 25 kg, intact mixed-breed male was brought to Evidensia Specialist Animal hospital Strömsholm, Sweden (ESVHS) in December 2023 (Table 1). The patient was originally from a dog shelter in Romania and had been imported to Sweden in April 2023 at an estimated age of five months. The dog had previously been diagnosed with pain of unknown origin at a local veterinary clinic, due to general stiffness, abdominal pain, fever and pain in the back and limbs. The clinical signs had improved after a seven-day course of doxycycline (Ronaxan, Boehringer Ingelheim) 10 mg/kg per os SID and robenacoxib (Onsior, Elanco) 1.7 mg/kg per os SID, but the dog relapsed once therapy was discontinued, and was subsequently referred to ESVHS.

**Table 1:** Chronologic summary of events for dogs suspected to be infected with *B. canis*

Date	Index dog				Contact dogs (n = 4)	
	Event	Clinical signs	Sampling	Test results	Sampling	Test results
2023						
April	Imported from Romanian shelter					
Summer		Pain, generalized stiffness				
Autumn	Examination by veterinarian	Severe back pain				
October	Doxycycline 10 mg/kg PO Robenacoxib 1.7 mg/kg PO					
December	Examination ESVHS	Severe back pain	Serology*	Pos		
			Urine culture^†^	Neg		
	Euthanasia Necropsy		Tissue culture^†^	Neg		
			Tissue PCR^‡^	Pos		
2024						
January					Serology*	1 Pos (C3) 3 Neg
					Urine culture^†^	All Neg
					Urine PCR‡	All Neg
February					Serology*	3 Pos (C1–C3) 1 Neg
					Urine culture^†^	All Neg
					Urine PCR‡	All Neg
April	Additional testing (frozen serum)		Serology**	Pos		
			Serology***	Pos		

Suspicion of brucellosis in index dog was raised based on clinical signs and the anamnestic information that the dog was imported from a shelter in a country where brucellosis occurs. The index dog was seropositive for *Brucella canis*, and positive for *Brucella* spp. on PCR. Three dogs from the same household (C1, C2, C3) were seropositive, of which one (C3) had a rising titre. One dog from same shelter (C4) was seronegative. Repeated selective culture for *Brucella canis* from both index dog and contact dogs were negative

*ESVHS* Evidensia Specialist Animal hospital Strömsholm, Sweden, *Pos* positive, *Neg* negative

*Enzyme-linked immunoassay (European Veterinary Laboratory, the Netherlands)

**Enzyme-linked immunoassay (Brucella ovis antibody test, VMRD Inc., USA)

***Lateral flow immunoassay (Antigen Rapid C. Brucella Ab Test Kit, BioNote, Inc., Republic of Korea)

^†^Non-selective media (horse blood agar, Bromo Cresol Purple Lactose agar, Trypticase-soy agar) and selective media (Farrell’s selective solid agar medium) at 37 °C in aerobic atmosphere

^‡^Real-time polymerase chain reaction targeting the IS711 insertion sequence of *Brucella* spp.

At ESVHS, a clinical examination revealed severe back pain, stiffness, lethargy and slightly enlarged testes. The rectal temperature was 39.6 °C. An abdominal ultrasound showed a mild to moderate prostatic pathology, mild generalised abdominal lymphadenopathy, and a heterogeneous appearance of the spleen. Further evaluation by radiography and computed tomography (CT) was suggested by the veterinarian but declined by the dog owner. Results from serum biochemistry and complete blood count were within reference values, apart from an elevated C-reactive protein of 40 mg/L (ref: <5). A SNAP 4Dx test (IDEXX Laboratories) for antibodies against *Borrelia burgdorferi*, *Anaplasma* spp. and *Ehrlichia* spp. as well as antigen of *Dirofilaria immitis* was negative, as was a PCR for *Ehrlichia* spp. The dog’s history and clinical presentation raised suspicion of brucellosis, prompting cystocentesis for urine culture and serum sampling for *B. canis* serology to be performed. A urine sample was sent to the Swedish Veterinary Agency (SVA) for *B. canis* culture on non-selective (horse blood agar, Bromo Cresol Purple Lactose agar, Trypticase-soy agar) and selective (Farrell’s selective solid agar medium) media at 37 °C in aerobic atmosphere [26, 27]. The culture was performed in a Biosafety level 3 laboratory and examined for growth after 3, 7 and 10 days. A serum sample was sent to European Veterinary Laboratory (EVL) in the Netherlands for analysis of *B. canis* antibodies by ELISA. Awaiting the results, analgesic treatment with pregabalin (Pregabalin, 1A pharma) 3 mg/kg per os BID and robenacoxib (Onsior, Elanco) 1.7 mg/kg per os SID was initiated. Culture revealed no bacterial growth, but serology detected presence of *B. canis* antibodies, with an IgG titre of 1:150 (cut off 1:100), strengthening the suspicion of brucellosis. The dog showed signs of consistent severe pain, despite analgesic therapy, and the owner decided to euthanise the dog. This decision was made based on a poor quality of life as the clinical signs did not respond to therapy, and an expected guarded prognosis, due to the lack of effective treatment against brucellosis.

The carcass was frozen and sent to SVA for necropsy. The main pathological findings pertained to the prostate, the spleen, the abdominal and deep iliac lymph nodes, and the vertebral column. The prostate displayed a mottled coloured pattern, with radiating red streaks from the urethra to the capsule. Moderate amounts of white fluid exuded from the prostate on cut surface. The spleen was mildly to moderately enlarged, with a fleshy texture and a follicular pattern. The lymph nodes in the abdominal cavity were mildly to moderately enlarged, as were, to a lesser extent, the lymph nodes in the thoracic cavity. The lymph nodes displayed several pinpoint sized white foci in the cortex. In the vertebral column a two-centimetre-wide spondylosis-like lesion was observed at L1–L2. The lesion was fully mineralised and could not be cut with a knife.

**Fig. 1:**
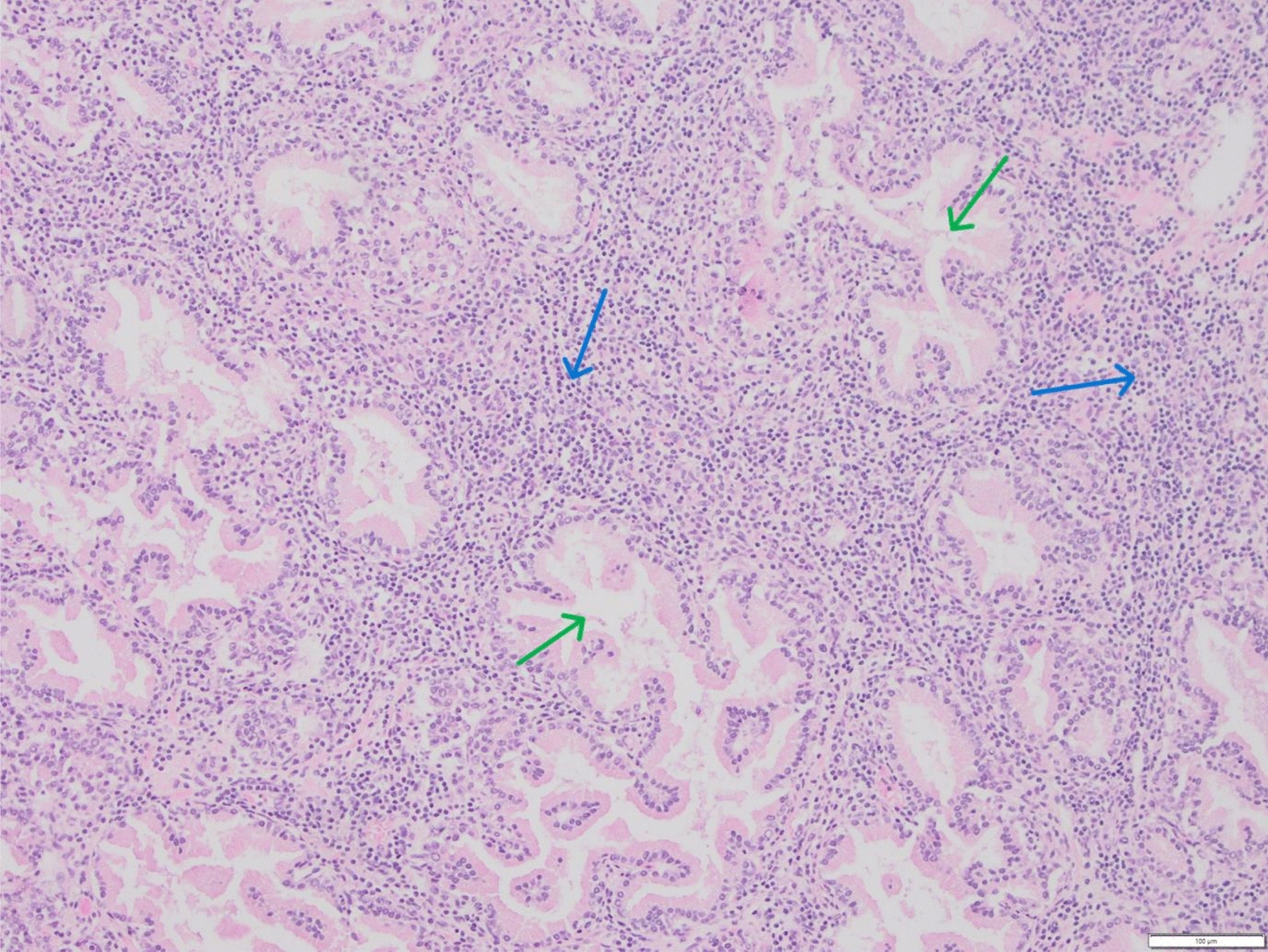
Photomicrograph showing histological section of the prostate, stained with haematoxylin and eosin, 10×. The interstitia are widened by a lymphoplasmacytic infiltrate (*blue arrows*), which separates the tubuloacinar glands (*green arrows*)

**Fig. 2:**
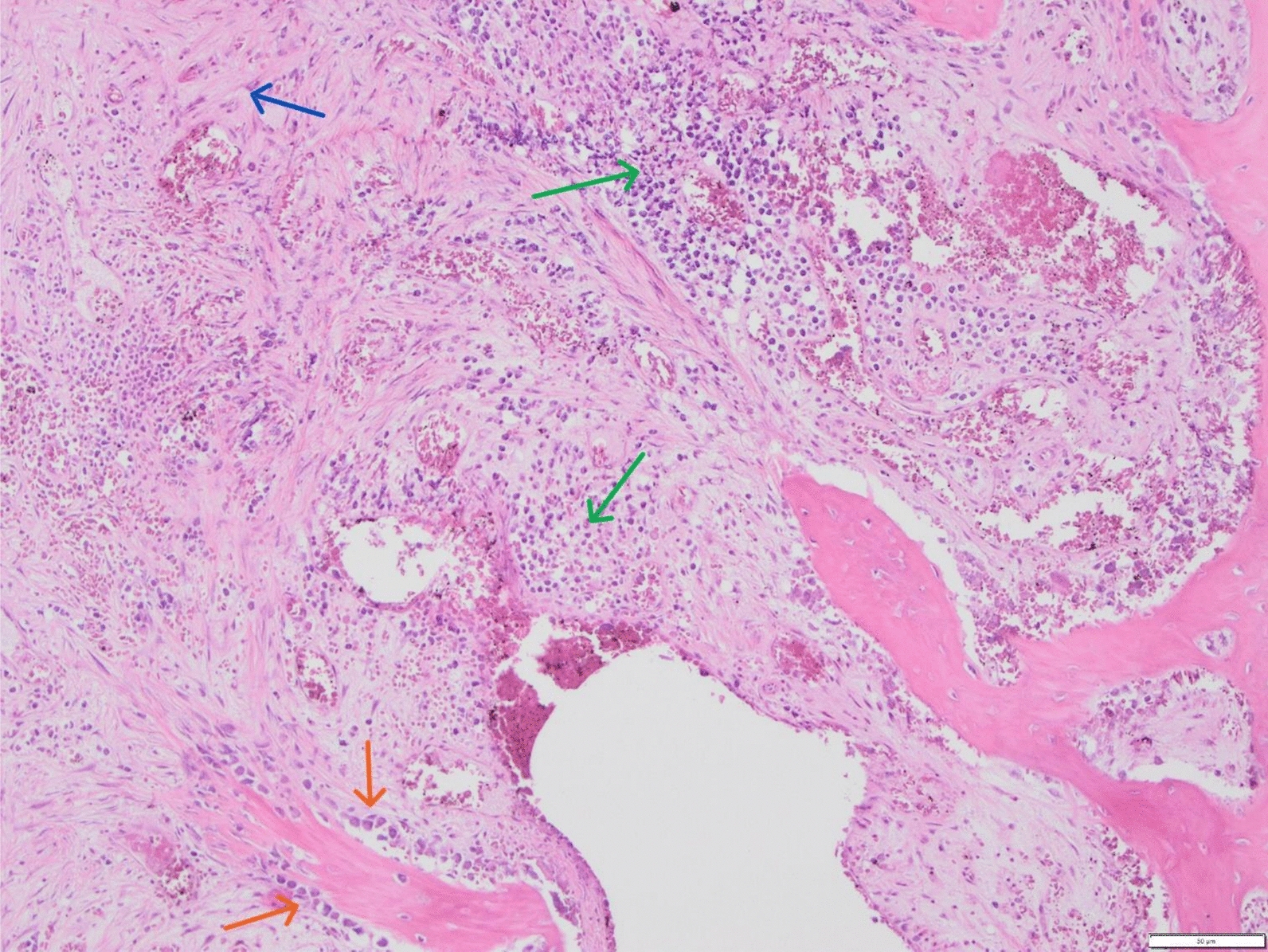
Photomicrograph showing histological section of the intervertebral disc, stained with haematoxylin and eosin, 20×. A degenerated bone trabeculae is surrounded by osteoblasts (*orange arrows*), a sign of regeneration. A mixed inflammatory infiltrate (*green arrows*) is seen in collagenous tissue. Collagenous tissue has replaced the space where lamellas of the disc should be (*blue arrows*)

Histopathologically, the prostate displayed inflammatory cell infiltrates, mainly lymphocytes and plasma cells, admixed with fewer numbers of histiocytes at sites of tubular destruction (Fig. 1). There was no granuloma formation in the prostate, testicles or epididymides. The spleen and lymph nodes showed signs of reactive hyperplasia. In the affected intervertebral disc, destruction of the normal lamellar architecture was observed, with replacement by collagenous tissue in varying stages of maturity, and granulation tissue. Mixed inflammatory cells and proliferation of osseus tissue was also seen (Fig. 2).

During necropsy, samples for culture and PCR were taken from four tissues: the prostate, testes/epididymis, deep iliac lymph node and spleen. Stamp’s modified Ziehl–Neelsen staining was performed with negative results. Culture from collected tissues was performed as previously described, with negative results. Two real-time PCR assays, targeting the IS711 insertion sequence of *Brucella* spp., were performed [28, 29]. The prostate was positive in both PCRs with Ct-values of 34–36, whereas the other samples were negative for *Brucella* spp.

After confirmation of infection by PCR, serum was analysed for *B. canis* antibodies by ELISA (Brucella ovis antibody test, VMRD Inc., USA), with a mean S/P ratio of 3.47 (cut off 1.47) and by a lateral flow immunoassay (Antigen Rapid C. Brucella Ab Test Kit, BioNote, Inc., Republic of Korea), with positive result.

**Fig. 3:**
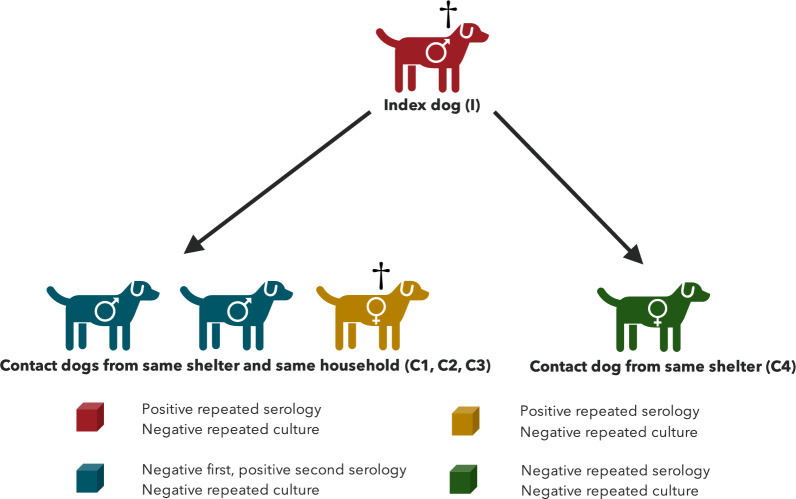
Schematic figure of index dog (I), with confirmed *Brucella canis* infection, and contact dogs (C1, C2, C3 and C4). Repeated selective culture from all dogs were negative. Index dog was seropositive for *Brucella canis*, and positive for *Brucella* spp. on PCR. C1, C2 and C3 from the same household were seropositive, of which C3 had a rising titre. C4 from same shelter was seronegative. ^†^Euthanised and sent for necropsy

The index dog (I) was housed together with one neutered male (C1) and two neutered female dogs (C2 and C3) (Fig. 3), all originating from the same Romanian shelter and imported to Sweden prior to the index dog. None of these dogs showed clinical signs of illness (Table 1). The three dogs were sampled for serology, urinary culture and PCR in January 2024, with a positive IgG-titre of 1:200 on ELISA (EVL, The Netherlands) for one of the females (C3). The other serological samples, the cultures (urine) and the PCRs (urine) were negative. Upon re-testing in February 2024, all dogs had a positive IgG-titre of 1:200, 1:300 and 1:500 respectively, the latter from the female with a previous positive titre (C3). Cultures (urine) and PCRs (urine) were again negative. The owners decided to euthanise the female dog that had the rising titres (C3) and the dog was subsequently sent to SVA for necropsy. Findings during necropsy consisted of mild enlargement of tonsils, submandibular lymph nodes and superficial cervical lymph nodes. Histological interpretation was limited by marked cadaverous changes. The findings were non-specific, with mildly enlarged lymphocytic follicles in the tonsils and lymph nodes (mild lymphadenitis and tonsilitis), as well as a moderate chronic inflammation and degenerative changes in the uterine remnant. Samples were taken from liver, spleen, lung, kidney, tonsil, joint capsule, and four different lymph nodes for selective *Brucella* culture and PCR for *Brucella* spp, which were all negative.

The index dog was found as a stray pup in Romania together with a female pup (C4), presumed to be a litter mate (Fig. 3). The female was imported to Sweden as an adult. Because of the findings in the index dog, this female was clinically examined and sampled for *B. canis* in January 2024. She showed no clinical signs of disease. Serological analysis by ELISA (EVL, The Netherlands) was negative and selective cultures (vaginal swab and blood) as well as two different real-time PCRs (vaginal swab) were also negative. She was re-tested in February 2024 with negative results on serology, selective cultures (urine, blood and vaginal swab) and PCR (urine, vaginal swab).

## Discussion and conclusions

This is the first described case of *B. canis* in a non-breeding dog in Sweden. The suspicion of brucellosis was based on the history and clinical signs, and it was strengthened further by the presence of antibodies against *B. canis*. The infection was confirmed postmortem by positive results with two different PCRs detecting *Brucella* spp. After PCR confirmation, two additional serological tests for *B. canis* were performed with positive results. This case highlights the complexity of diagnosing brucellosis in dogs, and that multiple diagnostic methods may be necessary to confirm an infection. It also acts as a reminder that importing shelter dogs from endemic countries may incur a risk of introducing the infection to non-endemic countries.

Despite a strong clinical suspicion of brucellosis in the index case, based on the origin of the dog and the clinical signs, the low serological titre could have been interpreted as a false positive result, especially since the urine sample was negative on culture. The explanation for the negative cultures of tissue samples taken at the time of necropsy of the index dog, despite the positive PCR result from prostatic tissue, could be a low bacterial load in the organs. This hypothesis is supported by the high Ct-values, indicating a low number of bacteria in the tissue. The bacterial growth might have been further suppressed by the antibiotic treatment and the fact that the carcass was frozen before necropsy. Postmortal decay also decreases the number of viable bacteria and can cause DNA to deteriorate. Both PCR assays used for diagnosis target the multi-copy insertion sequence IS711, which enables a high level of analytical sensitivity. Both assays have a reported specificity of 100%, and the limit of detection is 0.78 genomic equivalents and 2 fg, corresponding to approximately 0.56 genomic equivalents, respectively [28, 29]. Together, these data indicate that both assays are highly specific for *Brucella* and capable of detecting extremely small amounts of target DNA, even from non-viable cells, contributing to the overall assessment that the index dog was truly infected by *B. canis.*

Another explanation for the negative culture results could be that there were no viable bacteria left in the body at the time of euthanasia, as a result of a successful antibiotic treatment or immunological clearance. This is however less likely, since the common belief is that there is no satisfying treatment protocol for *B. canis* in dogs [14] and the dog was euthanised due to deteriorating clinical signs.

*Brucella canis* was discovered as an abortive agent in the 1960s, and the first studies focused on the reproductive changes in both males and females [3, 10, 30–33]. Enlarged lymph nodes were commonly described, but several studies emphasised the lack of fever or other systemic clinical signs [10, 30, 32]. Discospondylitis as a consequence of brucellosis has been described since the 1970s, and these dogs sometimes present with persistent or intermittent fever [4–6, 8]. In general, discospondylitis is of bacterial origin, most commonly caused by *Staphylococcus* spp., but sometimes other opportunistic bacteria [34]. Studies have identified features such as (1) younger age at diagnosis, (2) a chronic and slow progression of clinical signs, (3) involvement of section C1–C5, and (4) several discs affected, as more common in cases caused by *B. canis* than in cases caused by other bacteria, but the absence of these findings cannot be used to rule out *B. canis* [8, 35]. These criteria apply to the index dog, it being younger than one year at the onset of clinical signs as well as having clinical signs that were chronic and slowly progressing. However, only one disc was affected.

The positive serological results from the dogs in the same household (C1, C2, C3) indicate that they had been exposed to the infection, however the possibility of false positive results on serological tests should be considered. Infection of the contact dogs could not be confirmed by culture. If the dogs were truly infected, the most likely route of transmission would be through urine. The reported concentration of bacteria secreted in urine varies between studies, individuals and samplings. Even when a low bacterial burden is present, a continuous excretion and prolonged close contact has been proposed as the most likely route of transmission in groups of dogs without a history of abortions or possible venereal transmission [12, 36]. Excretion of bacteria in other body fluids, such as saliva and conjunctival fluid, is reported to be very low [36]. Several studies show that the shedding of bacteria in urine is higher in males than in females, with a persistent bacteriuria for at least 18 weeks [12, 36]. The contact dogs from the household had shared the environment with the index dog for several months.

For the contact dog that was found on the street as a pup (C4) together with the index dog, the suggested transmission would have been in utero or through their mother shortly after birth, if they were littermates. The suspicion of infection of this dog was dismissed based on the negative test results and absence of clinical signs. This suggests that they were in fact not littermates but merely found together. Bacterial shedding from infected pups is rarely described, instead transmission most often occurs after sexual maturation [12], which could explain why an infection could not be detected in this contact dog.

*Brucella canis* does not only cause disease and suffering in the individual dog. Suspected or confirmed infections in a dog can cause both emotional and financial suffering for the owner. The challenges of test result interpretations might further put ethical stress on the veterinarian. This by the balancing of the risk of failing to achieve a diagnosis, and thereby putting other dogs and humans at risk of exposure, and the risk of false positive results, which may lead to euthanasia on faulty grounds. Moreover, the zoonotic aspect must be considered. Direct contact with foetuses, abortive material and vaginal fluids is a common transmission route [37–39], but contact with dogs without a known involvement in parturition/abortion is also described [40, 41]. One case report described transmission of *B. canis* from a male pup to a child, indicating that bacterial spread might occur from dogs as early as at a few months of age [42]. However, experimental studies show that transmission from infected dogs usually starts when they reach sexual maturity [12]. A review on clinical human *B. canis* infections revealed that laboratory exposure counts for 20% of the described cases [43]. Most reports on human *B. canis* infections originate from the USA and a few other countries, including one publication from Europe [16, 38, 43], despite the fact that canine brucellosis is widespread. This could imply that *B. canis* in humans is underdiagnosed and underreported [16, 24, 43]. One explanation for this could be that the serological tests used in humans are developed for detection of antibodies against smooth *Brucella* spp., such as *B. melitensis* and *B. abortus*, rather than rough *Brucella* spp., such as *B. canis* [43]. Diagnosis in humans is therefore most often dependent on a clinical suspicion, followed by targeted investigations. A lack of knowledge and experience of the disease might decrease the suspicion rate [16, 40, 44].

Stray or shelter dogs with an unknown background might pose a risk for transmission of *B. canis* to humans and other dogs. The importance of information to veterinarians and animal health personnel, potential dog buyers and organisations importing these dogs is highlighted by several instances [14, 24]. The use of protective hygiene measures and communication about the risk of transmission to involved parties, such as dog owners and veterinary and laboratory personnel, should preferably be routine in all suspected cases.

In the UK, 49% (>38,000) of commercially imported dogs in 2021 originated from Romania, and this group is over-represented among suspected and confirmed cases of brucellosis [24]. As a consequence of the increase in cases of *B. canis* in dogs from Romania in the UK, requirements of a negative pre-import test result for dogs commercially imported from Romania came into force in October 2025 [45]. Such requirements are not available in Sweden, nor can they be applied within the European Union with the present legislation. There are some national recommendations for diagnostic testing of brucellosis, although not specific for import of shelter dogs, these are however not mandatory to follow. Illegally imported dogs are not included in the statistics from the Swedish Board of Agriculture. Between 200 and 530 dogs yearly have been identified at the Swedish border control as illegally imported between 2019 and 2021, but the true number of dogs smuggled into Sweden is likely much higher [46]. Since *B. canis* is more prevalent in stray dog populations [47–49], it is reasonable to suspect an increased prevalence of *B. canis* in smuggled dogs with an unknown background, as well as in dogs with a shelter background. The present case report support findings on *B. canis* in imported shelter dogs from Romania described in other studies and reports [14, 22–24].

This case report highlights the difficulties in recommended handling of dogs in contact with confirmed cases of *B. canis*. Seropositive dogs sharing the same environment as the index dog will in most cases be considered as exposed to the infection. Whether they truly are infected, and if so, to what extent they are at risk of spreading the infection is not known. Studies on risk of transmission from dogs without clinical signs, or dogs with non-reproductive clinical signs such as discospondylitis, are limited. Consequently, it is not possible to estimate the infectivity of the index dog in the present study, nor to be sure about the infectious status of the contact dogs. Knowledge on the pathogenesis of *B. canis* promotes the likelihood that excretion of the bacteria was intermittent, a plausible explanation of the negative urinary culture despite bacteria being identified in the prostate. If the index dog had not been sent for necropsy, a confirmatory diagnosis would not have been possible and the veterinarian would have been left with a presumptive diagnosis. There are probably other cases in Sweden, as well as in other countries, of dogs with strong suspicion of infection that cannot be confirmed by microbiological testing. In the absence of a definitive diagnosis, relevant precautionary measures to minimize the spread of infection should be considered in suspected cases. These measures could include neutering intact dogs, avoidance of close and prolonged contact with other dogs and with immunocompromised humans, young children and others at risk of infection, and prompt examination and targeted sampling if clinical signs arise in a suspected infected dog.

## Data Availability

The datasets used and analysed during the current study are available from the corresponding author on reasonable request.

## Declarations

### Prior publication

Data have not been published previously.
